# The Use of Concept-Mapping to Structure the Cultural Adaptation of Educational Curricula for Latino Older Adults

**DOI:** 10.1093/geroni/igab046.831

**Published:** 2021-12-17

**Authors:** Lissette Piedra, Melissa Howe, Yadira Montoya, Molly Hofer

**Affiliations:** 1 University of Illinois at Urbana-Champaign, Urbana, Illinois, United States; 2 NORC at the University of Chicago, Chicago, Illinois, United States; 3 University of Illinois Extension, Chicago, Illinois, United States

## Abstract

Culture, embedded in language and reflected in colloquial expressions, influences behaviors and cognitive constructs that affect health. To reach Latino older adults, health promotion efforts should include congruent cultural aspects—such as relevant metaphors, values, and proverbs—that will resonate with their cognitive constructs. However, this content should also be situated within a broader social context. For community-dwelling Latino older adults, this means considering their care systems and the multiple stakeholders within. In this paper presentation, we describe an innovative, interdisciplinary collaboration to culturally and linguistically adapt existing Illinois Extension curricula to meet the needs of Latino older adults and their families living in Cook County, which includes Chicago and its neighboring suburbs. We will demonstrate how concept-mapping (CM) studies can be used to structure the cultural adaptation of educational curriculum to a Latino audience. Specifically, we describe these CM studies, which asked how multiple stakeholders and Latino older adults living in the Chicagoland area defined positive aging provided empirically-grounded direction for our 11-member steering committee, composed of investigators, service leaders, and Latino older adults. We also will describe how the current project deepens relationships in the community that facilitate dissemination efforts to Latino older adults.

